# Functional characterization of DnSIZ1, a SIZ/PIAS-type SUMO E3 ligase from *Dendrobium*

**DOI:** 10.1186/s12870-015-0613-3

**Published:** 2015-09-17

**Authors:** Feng Liu, Xiao Wang, Mengying Su, Mengyuan Yu, Shengchun Zhang, Jianbin Lai, Chengwei Yang, Yaqin Wang

**Affiliations:** Guangdong Provincial Key Laboratory of Biotechnology for Plant Development, School of Life Sciences, South China Normal University, Guangzhou, 510631 China

**Keywords:** *Dendrobium*, Flowering time regulation, Stress responses, SUMO conjugates, SUMO E3 ligase, SUMOylation

## Abstract

**Background:**

SUMOylation is an important post-translational modification of eukaryotic proteins that involves the reversible conjugation of a small ubiquitin-related modifier (SUMO) polypeptide to its specific protein substrates, thereby regulating numerous complex cellular processes. The PIAS (protein inhibitor of activated signal transducers and activators of transcription [STAT]) and SIZ (scaffold attachment factor A/B/acinus/PIAS [SAP] and MIZ) proteins are SUMO E3 ligases that modulate SUMO conjugation. The characteristic features and SUMOylation mechanisms of SIZ1 protein in monocotyledon are poorly understood. Here, we examined the functions of a homolog of *Arabidopsis* SIZ1, a functional SIZ/PIAS-type SUMO E3 ligase from *Dendrobium*.

**Results:**

In *Dendrobium*, the predicted DnSIZ1 protein has domains that are highly conserved among SIZ/PIAS-type proteins. DnSIZ1 is widely expressed in *Dendrobium* organs and has a up-regulated trend by treatment with cold, high temperature and wounding. The DnSIZ1 protein localizes to the nucleus and shows SUMO E3 ligase activity when expressed in an *Escherichia coli* reconstitution system. Moreover, ectopic expression of DnSIZ1 in the *Arabidopsis siz1-2* mutant partially complements several phenotypes and results in enhanced levels of SUMO conjugates in plants exposed to heat shock conditions. We observed that DnSIZ1 acts as a negative regulator of flowering transition which may be via a vernalization-induced pathway. In addition, ABA-hypersensitivity of *siz1-2* seed germination can be partially suppressed by DnSIZ1.

**Conclusions:**

Our results suggest that DnSIZ1 is a functional homolog of the *Arabidopsis* SIZ1 with SUMO E3 ligase activity and may play an important role in the regulation of *Dendrobium* stress responses, flowering and development.

**Electronic supplementary material:**

The online version of this article (doi:10.1186/s12870-015-0613-3) contains supplementary material, which is available to authorized users.

## Background

In eukaryotic organisms, post-translational protein modification by methylation, phosphorylation, acetylation, glycosylation, or ubiquitination by Ub (ubiquitin) and Ubls (ubiquitin-like proteins), play important roles in diverse cellular regulatory processes [1, 2]. Similar to ubiquitination, Ubl modifications, such as SUMOylation, are known to facilitate reversible conjugation of a SUMO (small ubiquitin-related modifier) polypeptide to protein substrates by the formation of an isopeptide bond between the C-terminal glycine carboxyl group of SUMO and the ε-amino group of the lysine residue in the conserved SUMOylation sites of substrate proteins with the consensus motif (ψKXE/D; ψ, large hydrophobic residue; K, lysine; X, any amino acid; E, glutamate; D, aspartate) [1, 3–5]. SUMOylation through the SUMO conjugation pathway involves the sequential action of a series of enzymes: E1 SUMO activation enzyme, E2 SUMO conjugation enzyme and E3 SUMO ligase [3, 4, 6–9]. Subsequently, SUMO proteases can deconjugate SUMO from its associated substrate [1, 10–12]. Ubiquitination results in the targeting of the protein substrate for proteasomal degradation [13], whereas SUMOylation is a transient and reversible process that often results in an altered function and/or cellular localization of the modified protein [5, 10].

A number of studies have shown that SUMOylation is involved in a broad range of biological processes. For example, in animals and yeast, SUMO modifications affect cell cycle progression, DNA replication and repair, protein activity and stability, chromatin structural maintenance, subcellular localization and transcriptional regulation, as well as oxidative stress responses [4, 5, 10, 14–17]. SUMOylation in plants has been associated with biotic and abiotic stress responses, flowering and other aspects of development [18–24]. Moreover, protein SUMOylation by the *Arabidopsis thaliana* SUMO E3 ligase SIZ1 (AtSIZ1) has been shown to be involved in many environmental responses, including those related to phosphate deficiency, nitrogen assimilation, cell growth and development, tolerance of drought, cold, and high levels of copper, basal thermotolerance independent of the hormone salicylic (SA), SA-dependent signaling associated with both defense and development, flowering time regulation, and both auxin and abscisic acid (ABA) signaling [25–37]. Thus, plant post-translational modification by SUMOylation is of fundamental importance in modulating numerous signaling pathways.

SUMO E3 ligases participate in the SUMOylation pathways that are crucial to many eukaryotic biological processes. Several types of SUMO E3 ligases have been identified, including RanBP2 (RanGAP1-binding protein 2), Pc2 (Polycomb group 2), NES2/MMS21 (non-SMC element/methyl methanesulfonate sensitive 1) and SIZ/PIAS (SAP and MIZ/protein inhibitor of activated STAT) [14, 38–42]. Of these, SIZ/PIAS family proteins, which are characterized by an essential SP-RING domain, represent the largest group of SUMO E3 ligases, all of which share high degree of sequence identity. SIZ/PIAS E3 ligases have five structural motifs: an N-terminal SAP (scaffold attachment factor A/B/acinus/PIAS) motif, a PINIT (Pro-Ile-Asn-Ile-Thr) motif, a SP-RING zinc finger domain, a SXS (for serine-X-serine, where X is any amino acid) domain and a PHD motif (plant homeodomain) [30, 43–46]. There are two PIAS-type SUMO E3 ligases that have been identified in *Arabidopsis* are SIZ1 [25, 30] and MMS21/HPY2 (NSE2/MMS21-type High Ploidy 2) [47–49].

Most studies to date of plant E3 ligases have focused on those of the experimental model *A. thaliana* and comparatively little is known about these proteins in agronomically- and horticulturally-important species. In this report, we describe the characterization of DnSIZ1 [50], a functional SUMO E3 ligase from the monocot *Dendrobium*, the largest genus of the Orchidaceae, which contains species that are mainly distributed in tropical and subtropical regions. The Orchidaceae are one of the largest families of flowering plants and many members of this family have high ornamental and commercial value. Here we report the activity and subcellular localization of DnSIZ1, together with an assessment of its expression patterns and function *in planta* through overexpression in transgenic *Arabidopsis* lines. Our results suggest that DnSIZ1 is a functional homolog of the *Arabidopsis* SIZ1 and may play an important role in the regulation of *Dendrobium* stress responses, flowering and development.

## Results

### Molecular characterization of *DnSIZ1*

**Fig. 1:**
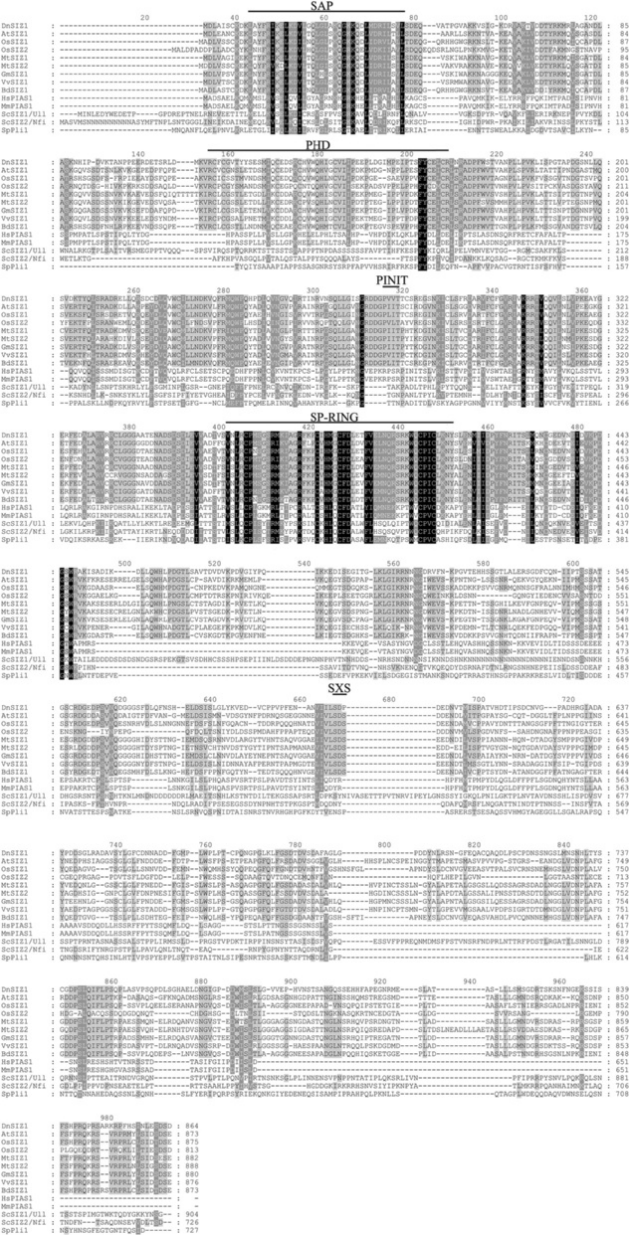
Amino acid sequence alignment of the SIZ/PIAS proteins. The deduced amino acid sequence of DnSIZ1 was aligned with the amino acid sequences of SIZ/PIAS homologs from other species. The sequences were obtained from the NCBI protein database using the BLAST network service. Amino acid sequences of SIZ/PIAS proteins from *Dendrobium* (Dn), *Arabidopsis thaliana* (At), *Oryza sativa* (Os)*, Medicago truncatula* (Mt), *Glycine max* (Gm), *Vitis vinifera* (Vv), *Brachypodium distachyon* (Bd), *Homo sapiens* (Hs), *Mus musculus* (Mm), *Saccharomyces cerevisiae* (Sc) and *Schizosaccharomyces pombe* (Sp). The domains include: the SAP (Scaffold attachment factor A/B/acinus/PIAS); the PHD (Plant homeodomain); the PINIT (Pro-Ile-Asn-Ile-Thr); the SP-RING (SIZ/PIAS-RING); and the SXS (Ser-X-Ser) domain. Black and gray shaded backgrounds indicated amino acids that were identical residues or conservative substitutions, respectively. Hyphens indicated gaps introduced to optimize alignments. Numbers above the alignment indicated the number of the amino acids from the first amino acid

**Fig. 2:**
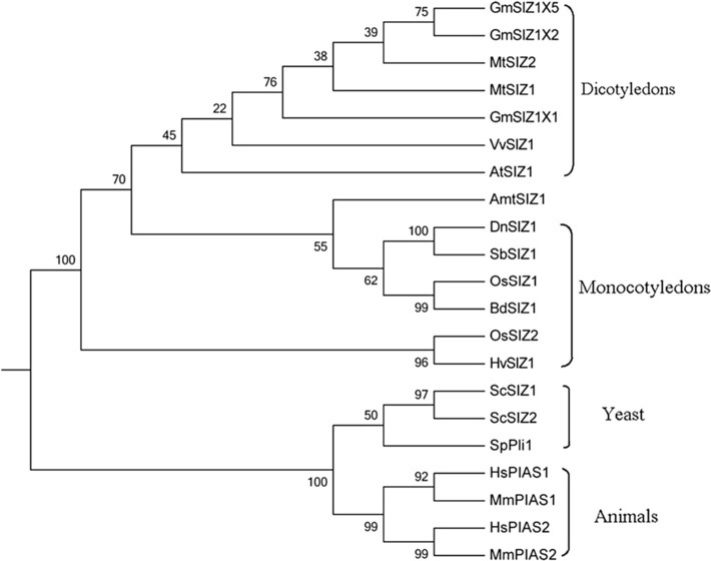
Phylogenetic analysis of SIZ/PIAS proteins between *Dendrobium* and other species. The species are *Glycine max* (Gm), *Medicago truncatula* (Mt), *Vitis vinifera* (Vv), *Arabidopsis thaliana* (At), *Amborella trichopoda* (Amt), *Dendrobium* (Dn), *Sorghum bicolor* (Sb), *Oryza sativa* (Os)*, Brachypodium distachyon* (Bd), *Hordeum vulgare* (Hv), *Saccharomyces cerevisiae* (Sc), *Schizosaccharomyces pombe* (Sp), *Homo sapiens* (Hs) and *Mus musculus* (Mm). The complete protein sequence were used to construct the phylogenetic tree with MEGA 6.06 software and the Maximum Likelihood method [83]. The numbers at the nodes indicated the bootstrap values. Bootstrap testing was performed with 1000 resamplings

In *Arabidopsis*, the PIAS-type SUMO E3 ligase AtSIZ1 regulates responses to hormones, abiotic and biotic stresses, and controls flowering [26, 27, 30, 31, 34, 37, 45]. We previously reported the isolation of *DnSIZ1*, the *Dendrobium* homolog of *AtSIZ1* [50]. The *DnSIZ1* gene encodes protein of 864 amino acids and its nucleotide sequence was deposited in GenBank (KT375328). To investigate the biological roles of DnSIZ1, we first identified and aligned the sequences of SIZ/PIAS family proteins from the NCBI protein database. The results indicated that the DnSIZ1 deduced protein has three predicted domains (SAP, SP-RING and plant-specific PHD domains), which are conserved in AtSIZ1 [30] (Fig. 1). In addition, a Pro-Ile-Asn-Ile-Thr (PINIT) core motif was identified that is more similar to the equivalent sequence in rice (*Oryza sative*) [51]. But the SXP domain is different from SXS domain in *Arabidopsis* and rice. In plant SIZ/PIAS-type proteins there are five conserved domain structures: SAP, PHD, PINIT, SP-RING and SXS [30]. The SAP domain, which is specially required for trans-repression activity of PIAS, can form a helix-extended loop-helix structure that binds to DNA [52]. The PHD domain, which is only present in plant SIZ/PIAS homologs, is a C4HC3 Zn-finger [53], while the PINIT and SP-RING domains are essential for the SUMO E3-ligase activity of SIZ/PIAS proteins [54]. Based on the protein sequence alignment and the phylogenetic relationships among DnSIZ1 and homologous proteins from other species (Figs. 1 and 2), DnSIZ1 shares high sequence identity with SIZ/PIAS proteins of other species in regard to conserved structures that are essential for SUMO E3 ligase activity and are especially similar to SIZ1 proteins in rice and sorghum.

### Expression patterns of *DnSIZ1*

**Fig. 3:**
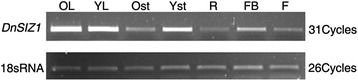
The expression patterns of *DnSIZ1* gene. RT-PCR analysis of *DnSIZ1* transcripts in different organs of *Dendrobium*. 18sRNA served as an internal control. OL, old leaves; YL, young leaves; Ost, old stem; Yst, young stem; R, root; FB, flower bud; F, flower

**Fig. 4:**
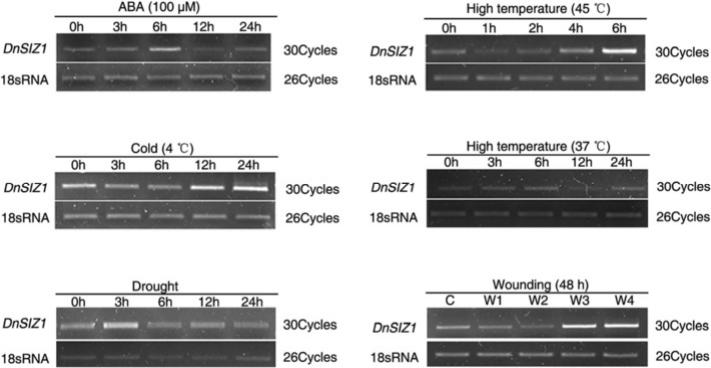
Expression of *DnSIZ1* in response to stress treatments. The stresses include: ABA (100 μm), cold (4 °C), drought, high temperature (37 °C, 45 °C), and wounding. Seedlings were collected at various time intervals after the start of the stress treatment (t = 0). 18sRNA served as an internal control

To gain insights into the potential functions of *DnSIZ1*, we analyzed the temporal and spatial expression patterns of its transcript accumulation using semi-quantitative RT-PCR. As shown in Fig. 3, transcripts of *DnSIZ1* were detected in all organs, including roots, stems, leaves, flowers and flower buds, with the higher expression level being detected in old leaves, young leaves and young stems, and lower expression in roots and flowers. Next, we analyzed whether the expression of *DnSIZ1* gene could be induced in response to abiotic and hormonal stress. The results showed *DnSIZ1* expression was reduced first and strongly up-regulated later when treated with high temperature (45 °C), cold (4 °C) and wounding (Fig. 4).

### Subcellular localization of DnSIZ1

**Fig. 5:**
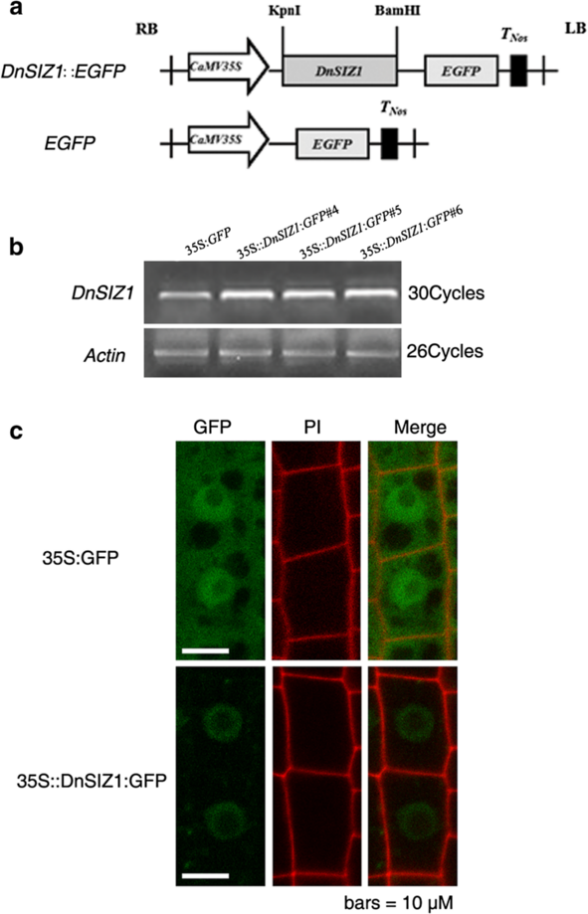
Subcellular localization of DnSIZ1 protein. **a** Schematic representation of constructs for constitutive expression in *Arabidopsis* root cells. *CaMV35S*, c*auliflower mosaic virus 35S* promoter; EGFP, green fluorescent protein; *T*_*Nos*_, nopaline synthase gene terminator are indicated. **b** DnSIZ1 amplification and identification of transgenic *Arabidopsis* lines. The plasmids were transformed into *Arabidopsis* wild-type using an *A. tumefactions*-mediated floral dip method. **c** Co-expression of GFP fused to DnSIZ1. *35S*::*DnSIZ1*:*GFP #4* and *35S*:*GFP* transgenic *Arabidopsis* root cells were observed, respectively. Signals were visualized using a confocal laser scanning microscopy. Panels from left to right: GFP fluorescence image; propidium iodide stained image; merged GFP fluorescence image. The GFP green color (Merged) revealed that DnSIZ1 is localized to the nucleus. Scale bars = 10 μm

Previous studies showed that *Arabidopsis* SIZ1 is localized to the nucleus and accumulates in nuclear speckles [25, 45]. To determine the subcellular localization of the DnSIZ1 protein, we expressed the GFP fusion constructs in wild-type (Col-0) *Arabidopsis* roots, the line *35S*::*DnSIZ1*:*GFP #4* was used (Fig. 5a, b). The fluorescent signal was monitored by confocal microscopy and it was determined that the fusion protein accumulated in the nucleus (Fig. 5c).

### Phenotypic analysis of *DnSIZ1* transgenic plants

**Fig. 6:**
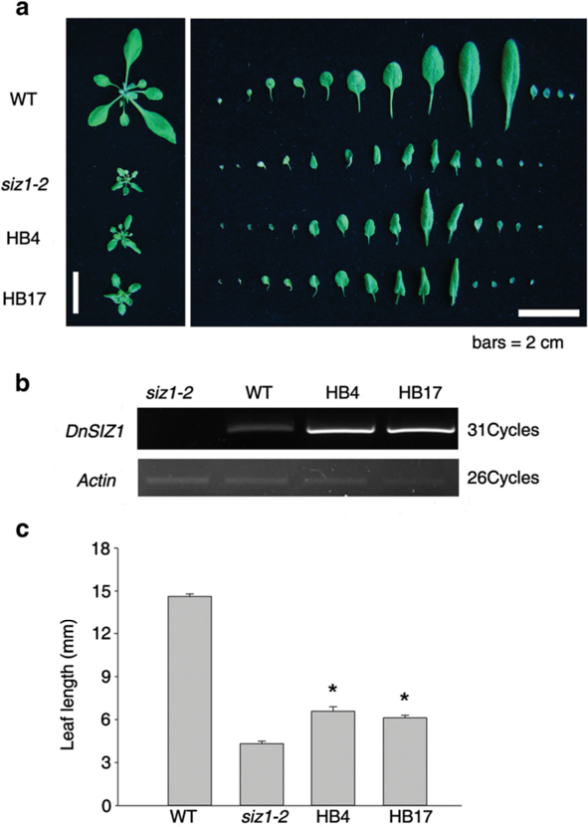
Phenotypic analysis of *DnSIZ1* overexpressing plants and their corresponding expression levels. **a** Leaf phenotypes of plants expressing *DnSIZ1*. The leaves were taken and arranged in each row from the left. Plants expressing *DnSIZ1* in the *siz1-2* (*atsiz1-2*) mutant were compared with Col-0 and *siz1-2*. **b** Expression of *siz1-2* (*atsiz1-2*) plants expressing *DnSIZ1*. Total RNA was extracted from three-week-old plants and RT-PCR was performed using gene-specific primers (*DnSIZ1-*F, *DnSIZ1-*R). *Actin* was used as an internal control. **c** The results of statistical analysis of leaf length for each of the lines are shown in (**a**). The largest leaves of each line were used for leaf length measurement. The data are the means of three different experiments and indicate the percentage (± SE) of leaf length in each transgenic plant line. Significant differences from *siz1-2* (asterisks) at *P* < 0.05 are indicated. Bar = 2 cm

**Fig. 7:**
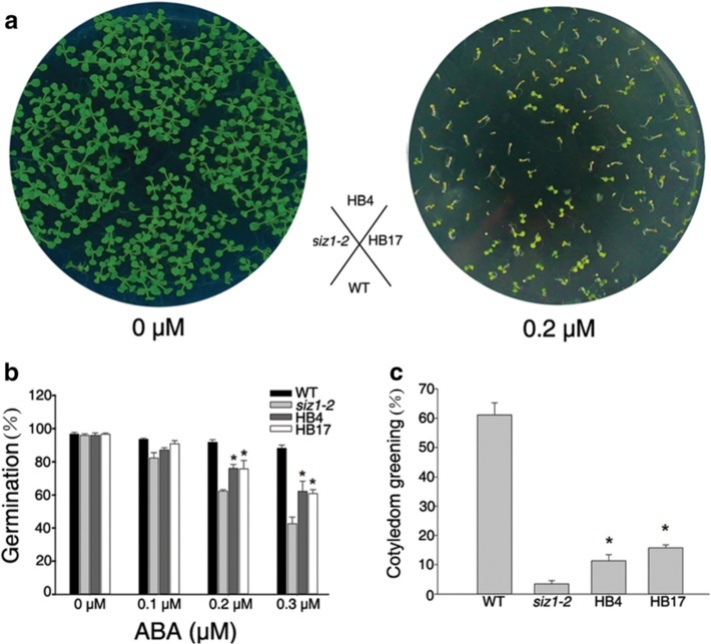
Inhibition of *siz1-2* ABA-hypersensitivity by overexpression of *DnSIZ1*. Seeds of wild-type (col-0), *siz1-2* (*atsiz1-2*), and each of the transgenic lines (HB4, HB17) were sown on MS medium without or supplemented with ABA. **a** Cotyledon expansion of transgenic plants in the presence of ABA. Stratified seeds of Col-0, *siz1-2* (*atsiz1-2*), and each of the transgenic lines (HB4, HB17) were spread in the illustrated pattern on MS medium containing 0 and 0.2 μM ABA and maintained under a 16 h-light/8 h-dark conditions at 22 °C. **b** Germination rates of seedlings 48 h after stratification. **c** Cotyledon expansion of 5 day-old seedling in the presence or absence of 0.2 μM ABA. The data represent the averages of three independent experiments. The percentages (±SE) of seedlings with green cotyledon in each genotype are shown. Significant differences from *atsiz1-2* (asterisks) at *P* < 0.05 are indicated

To further understand the function of *DnSIZ1*, it was overexpressed in the *Arabidopsis siz1-2* mutant. The *siz1-2* phenotypes of dwarfism with leaf curl and short leaf length were partially functionally rescued in plants from two separate transgenic lines (HB4, HB17) (Fig. 6). For example, the length of the largest leaves of the DnSIZ1 transgenic plants was 42 – 54 % greater than the equivalent leaves of the untransformed mutant. In addition to the stunted phenotype, *siz1-2* plants are hypersensitive to exogenous ABA [33]. To test whether DnSIZ1 overexpression can functionally complement the ABA hypersensitive phenotype of *siz1-2*, we investigated the ABA responses of the different genotypes. We also observed that the seed germination rates of the *DnSIZ1* transgenic lines were strongly enhanced relative to those of *siz1-2*, although slightly lower than those of wild-type plants at 48 h after stratification (Fig. 7a, b). Furthermore, analysis of cotyledon greening showed that the DnSIZ1 transgenic lines were less sensitive to ABA than *siz1-2* when grown on media containing 0.2 μM ABA, although they were more sensitive than wild-type plants (Fig. 7c). Taken together, these results indicate that DnSIZ1 can partially complement at least some of the functions of AtSIZ1 in development and ABA signaling pathways.

### *DnSIZ1* negatively regulates flowering time

**Fig. 8:**
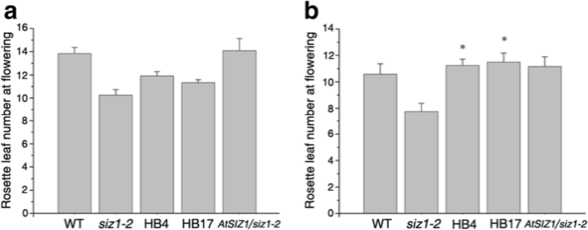
Comparison of the flowering time of wild-type, *siz1-2*, *35S*::*DnSIZ1* and *35S*::*AtSIZ1* transgenic plants. **a** Seeds of the four genotypes were stratified for 2-3 d and then grown in the greenhouse under long-day conditions. **b** Seeds of the four genotypes were stratified for 3 weeks and then grown in the greenhouse under long day conditions. The flowering time was estimated based on the number of rosette leaves as described above [31]. The data are averages of three independent experiments. Values presented are the percentages of rosette leaf numbers in transgenic plant line (±SE). Significant differences from *atsiz1-2* (asterisks) at *P* < 0.05 are indicated

Flowering marks the transition from vegetative maturity to the reproductive stage of development [55], and the floral transition is mainly regulated by photoperiod, vernalization, the autonomous pathway and gibberellin (GA)-dependent signaling [56, 57]. The time needed for the transition from vegetative development to reproductive growth of wild-type, *siz1-2* and *DnSIZ1* transgenic plants were recorded by counting the numbers of rosette leaves at the time of flowering [31]. Under normal growth conditions, rosette leaf numbers at flowering in the *DnSIZ1* transgenic plants relative to *siz1-2* were slightly higher, but were lower than those of wild-type and *AtSIZ1* transgenic plants (Fig. 8a). However, following a vernalization treatment at 4 °C for three weeks, rosette leaf numbers at flowering of all genotypes decreased. But, rosette leaf numbers at flowering of *DnSIZ1* transgenic plants are significantly more than those of *siz1-2* mutant (Fig. 8b). Thus, the early flowering phenotype of *siz1-2* was suppressed by overexpressing *DnSIZ1* gene following vernalization, indicating that *DnSIZ1* may repress flowering through vernalization-induced transition to flowering.

### Heat shock-induced accumulation of SUMO conjugates in *DnSIZ1* transgenic lines

**Fig. 9:**
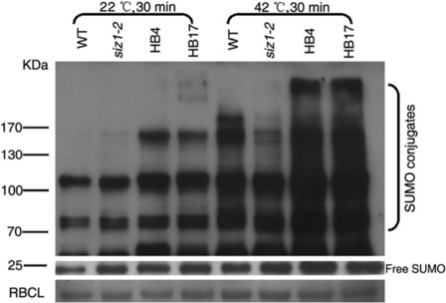
Heat shock-induced accumulation of SUMO conjugates in transgenic lines. Total proteins were extracted from untreated (22 °C, 30 min) or heat-shocked (42 °C, 30 min) 10 d-old seedlings of the wild type (Col-0), *siz1-2* (*atsiz1-2*), and each of transgenic lines (HB4, HB17). 20 μg of proteins was on an SDS-PAGE gel and the immunoblot was probed with an anti-SUMO1 antibody

Previous studies have shown that heat shock can induce SUMOylation [58], SUMOylation in wild-type *Arabidopsis* and *OsSIZ1/OsSIZ2* heterogenous transgenic lines are stronger than those in *Arabidopsis siz1-2* plants [26, 51]. In our study, the DnSIZ1 protein also participated in response to heat shock-induced accumulation of SUMO conjugates. Under normal conditions, SUMO conjugates in wild-type, *siz1-2* and *DnSIZ1* overexpressing transgenic *Arabidopsis* plants accumulated at relatively low levels. However, when exposed to a 42 °C heat shock for 30 min, transgenic lines produced significantly greater amounts of SUMO conjugates than *Arabidopsis siz1-2* plants (Fig. 9), suggesting that DnSIZ1 can functionally complement *Arabidopsis siz1-2* in the SUMO conjugation pathway. Collectively, these results suggested that the DnSIZ1 protein exhibits SUMO E3 ligase activity that can contribute to the SUMO modification pathway.

### DnSIZ1 is a functional SUMO E3 ligase

**Fig. 10:**
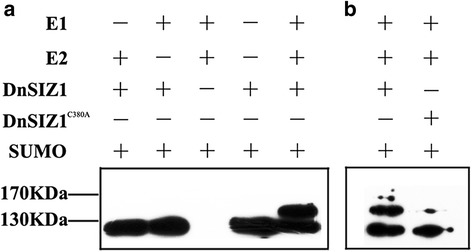
*In vitro* assays indicate that DnSIZ1 is a functional SUMO E3 ligase. **a** pCDFDuet-1*-*Flag-DnSIZ1 was expressed in *Escherichia coli* and then tested for SUMOylation activity in the presence of E1 (His-AtSAE1a-AtSAE2), E2 (His-AtSCE1) and Myc-SUMO1. **b** pCDFDuet-1*-*Flag-DnSIZ1 and pCDFDuet-1*-*Flag-DnSIZ1^C380A^ were expressed in *Escherichia coli* and then tested for SUMOylation activity. Immunoblots generated from these samples were probed with anti-Flag antibodies

In order to further demonstrate the ability of DnSIZ1 to act as a SUMO E3 ligase, we generated *pCDFDuet-1-Flag-DnSIZ1* and *pCDFDuet-1-Flag-DnSIZ1*^*C380A*^ constructs, in which the C380A mutation was introduced in its SP-RING, that is conserved in SIZ1s in different species and essential for SUMO ligase activity [45]. Then we performed SUMOylation assays in *E.coli* strain expressing DnSIZ1 together with AtSAE1a-SAE2, AtSCE1a and AtSUMO1 [59, 60]. Compared with negative control, in the presence of AtSAE1a-SAE2, AtSCE1a and AtSUMO1, SUMOylation of DnSIZ1 was detected, indicating that DnSIZ1 can be sumoylated by E2 SUMO conjugation enzyme, which is a characteristic feature of SUMO E3 ligase (Fig. 10a). In addition, the sumoylated band of DnSIZ1^C380A^ is weaker than that of DnSIZ1, which suggested the SP-RING is important for SUMOylation of DnSIZ1, providing envidence to support it is a potential SUMO E3 ligase (Fig. 10b). Coomassie brilliant blue staining of total protein was used as the loading control shown in Additional file 1: Figure S1.

## Discussion

SUMOylation is a post-translational regulatory process in eukaryotes. In plants, SUMOylation has mostly been studied in *Arabidopsis*, where it is involved in ABA responses, flowering time, phosphate starvation responses, salicylic acid (SA)-dependent defense responses, cold tolerance, drought, basal thermotolerance and removal of heavy metal [25, 26, 31, 35, 37, 49, 61–64]. In the context of *SIZ* gene functions, among the monocotyledons only *SIZ1* and *SIZ2* from rice have been described and it has been suggested that they functionally complement *Arabidopsis AtSIZ1* in the SUMO conjugation pathway [51]. To date little is known about the biological significance and SUMOylation mechanism in the ornamental monocotyledon *Dendrobium*. In this current study, a *Dendrobium* SIZ gene, *DnSIZ1*, was identified based on homology to SIZ genes from rice, sorghum and *Arabidopsis* [50]. The SAP, SP-RING and PHD domains of DnSIZ1 have a high degree of sequence conservation with those of AtSIZ1, while the PINIT domain is most similar to that of the OsSIZ2. However, in the SXS domain, the amino acid sequence is SXP instead of SXS, which we propose represents a species specific change (Fig. 1). Given that DnSIZ1 has high degree of sequence identity with SIZ1 proteins from rice and sorghum (Fig. 2) and has the canonical SP-RING domain that is necessary for the SUMO E3 ligase activity of SIZ/PIAS proteins [43], we speculate that *DnSIZ1* encodes an SP-RING protein with SUMO E3 ligase activity.

The expression pattern of *DnSIZ1* gene was shown in Fig. 3. *DnSIZ1* transcripts were detected in all tissues tested, and the expression was higher in leaves and young stems than other tissues. Evaluation of the expression in response to various environmental and hormonal stimuli (Fig. 4) showed that *DnSIZ1* expression had an up-regulation trend in response to high temperature (45 °C), cold (4 °C) and wounding. Not only significant transcripts were induced in response to high temperature, but also SUMO conjugates increased obviously in *DnSIZ1* transgenic plants after heat treatment (Fig. 9). Thus, it is likely that the activity of DnSIZ1 might be controlled at the transcriptional level and post-translational level in response to heat stress [51]. We also noted that the accumulation of SUMO conjugates in *siz1-2* remained inducible by heat treatment, but the increase of SUMO conjugates in *DnSIZ1* transgenic plants was highly dependent on DnSIZ1 activity, since the accumulation of SUMO conjugates was significantly lower in *siz1-2* compared with *DnSIZ1* transgenic plants (Fig. 9). Many reports showed that SUMOylation pathway member E3 ligase is dramatically affected by heat stress [65, 66]. Moreover, some SUMO E3 ligases such as AtSIZ1, OsSIZ1/OSSIZ2 and HPY2 can also improve thermotolerance in plants [26, 48, 51, 64]. Our results imply that DnSIZ1 plays a general role in heat stress response and may enhance heat tolerance in plants.

Although we have established that key domains of DnSIZ1 are conserved in the AtSIZ1 protein, we wanted to further confirm that DnSIZ1 can function as a SUMO E3 ligase. Accordingly, we constructed a SUMOylation reactions system in *E.coli* [59, 60] and demonstrated that DnSIZ1 is a functional SUMO E3 ligase (Fig. 10a, b). In addition, we observed the subcellular location of DnSIZ1 and the fluorescent signal in roots derived from the transgenic plants showed that DnSIZ1 protein accumulated in the nucleus (Fig. 5), which is further evidence for DnSIZ1 function as a SUMO E3 ligase in *Dendrobium*. It is significant because many transcription factors can be targeted by SUMO conjugation mediated by SIZ/PIAS proteins [15, 67]. For example, the transcription factors PHR1, ICE1, ABI5, MYB30 and FLC have been identified as targets of SIZ1 in *Arabidopsis*. These transcription factors are involved in phosphate starvation responses, cold tolerance, ABA responses and flowering time [25, 29, 31–33, 68, 69]. Taken together, our results indicate that the DnSIZ1 protein is localized in the nucleus, may regulate transcription factors in response to a variety of environmental stresses through SIZ1-dependent SUMO conjugation.

The *Orchidaceae* is the largest family in the plant kingdom and *Dendrobium* is a sizeable genus consisting of more than a thousand species that are native to South Asia, Australia, New Zealand, and Oceania, many of which show evidence of adaptation to stresses imposed by their environments, such as nutrient starvation, heat, cold, drought and wounding [70]. While *Dendrobium* spp. are valued as ornamental plants worldwide, traditional breeding methods of sexual hybridization and selection are too time-consuming to meet the increasing global demand. One major obstacle for *Dendrobium* breeding is the prolonged vegetative phase before *Dendrobium* switches to reproductive development [71]. Thus, the study of flowering transition and elucidation of the underlying molecular mechanisms in *Dendrobium* is of great potential commercial value. Broadly speaking, five genetically defined pathways have been identified that control flowering: the vernalization pathway, the photoperiod pathway, the gibberellin-mediated pathway, the autonomous pathway and the endogenous pathway [72]. Previous studies have shown that AtSIZ1 acts as a negative regulator of the transition to flowering through mechanisms that reduce SA accumulation and involve SUMO modification of *FLOWERING LOCUS D (FLD)* and *FLOWERING LOCUS C(FLC)* [31, 69, 73]. In this current study, we found that DnSIZ1 may repress flowering through vernalization-induced floral transition (Fig. 8). Vernalization-induced flowering is an adaptation to cold conditions in many species, but most of what is known about the molecular mechanisms underlying vernalization has resulted from studies of *A. thaliana* [74]*.* In *Dendrobium*, flowering time is induced by low temperatures [75]. Moreover, *DnSIZ1* expression strongly responded to cold (4 °C) (Fig. 4). It implies the negative regulation of the flowering transition by DnSIZ1 may operate through the vernalization pathway, but the exact molecular mechanisms are still unclear because no orthologs of *Arabidopsis FLC* have been found in *Dendrobium* so far. We note that this characteristic of *DnSIZ1* may have practical applications for enhancing the economic value of this ornamental crop.

The *Arabidopsis siz1* mutant shows a dwarf phenotype with leaf curling and short leaves [25, 27, 28, 34] and we used these characteristics to assess functional conservation of DnSIZ1. To this end we overexpressed *DnSIZ1* under the control of the *CaMV35S* promoter in the *siz1-2* mutant and observed that the mutant phenotypes were indeed partially functionally complemented (Fig. 6). Moreover, *DnSIZ1* overexpression partially rescued the ABA hypersensitivity of *siz1-2* in seed germination stage (Fig. 7). These results show DnSIZ1 may play an important role in plant development. Taken together, the biological functions of DnSIZ1 are multiple and complex, similar to *Arabidopsis* SIZ1. In order to further explore the biological functions of *DnSIZ1*, we are now establishing a *Dendrobium* transformation system to suppress *DnSIZ1* expression using RNAi technology. In addition, the exact molecular mechanisms by which DnSIZ1 controls flowering time as well as the regulatory network of SUMOylation will be the target of future studies.

## Conclusions

We have characterized the biological functions of DnSIZ1, a functional SIZ/PIAS-type SUMO E3 ligase in *Dendrobium*, and the results indicated substantial evolutionary conservation with, and a similar biological function to *Arabidopsis* AtSIZ1. The DnSIZ1 protein localizes to the nucleus and has SUMO E3 ligase activity when assayed in *E.coli* recombinant system and modulated by heat stress condition. Moreover, *DnSIZ1* overexpression can partially complement several *Arabidopsis siz1-2* mutant phenotypes. The expression of *DnSIZ1* gene is detectable in all organs and can be induced by various stress conditions. Overall, DnSIZ1 is a functional SUMO E3 ligase and play an important role in the regulation of *Dendrobium* stress responses, flowering and development.

## Methods

### Plant materials and growth conditions

The *Arabidopsis thaliana* wild-type (WT) and the *siz1-2* (SALK_065397) mutant [25] used in this study were the Columbia-0 (Col-0) background. *Arabidopsis* seeds were surface sterilized and plated on 1 × Murashige and Skoog (MS) medium containing 1.5 % sucrose, 0.8 % agar, pH 5.7 and then stratified for 2–3 d at 4 °C. Seedlings grown in plates or soil were incubated in a greenhouse at 22 °C under a 16 h-light/8 h-dark photoperiod, with a light intensity of 100 μmol m^−2^ s^−1^ and 60–80 % relative humidity. Plants of *D. nobile* were provided by the Guangdong Key Laboratory of Biotechnology for Plant Development (Guangzhou, China). The plants were grown in a greenhouse under natural light conditions. The temperature ranged from 23 to 27 °C and the relative humidity was 70 %.

### Vector construction and plant transformation

For preparing *DnSIZ1 A. thaliana* overexpression lines, the full-length *DnSIZ1* cDNA was amplified from *Dendrobium* cDNA library by RACE and RT-PCR [50]. The cDNA was cloned into a pMD18-T vector (TaKaRa, Japan) and verified by DNA sequencing. The plasmid DNA harboring the full-length fragment of *DnSIZ1* was digested with *Kpn*I and *Spe*I prior to subcloning the *DnSIZ1* fragment into the corresponding sites of the binary vector pCAMBIA1300-221, which contains that *CaMV35S* promoter. The constructed plasmid was transformed into the *Arabidopsis siz1-2* mutant following the *Agrobacterium tumefaciens*-mediated floral dipping transformation method [76]. Transgenic seedlings were obtained by antibiotic selection on MS agar plates and then semi-quantitative RT-PCR analysis was used to confirm the presence of the gene. Homozygous T3 or T4 generation plants from two independent transgenic lines (HB4, HB17) were used for phenotypic analysis and subsequent experiments. To generate the *35S*::*DnSIZ1*:*GFP* fusion construct, the entire coding region of *DnSIZ1* was inserted directly upstream of the EGFP (enhanced green fluorescent protein) coding region in pBEGFP (pBEGFP was reconstructed based on pBin19). The constructed plasmid was transformed into the Col-0 and T2 generation plants were used for subcellular localization analysis. Primer sequences for gene amplifications are listed in Additional file 2: Table S1.

To generate the constructs expressing AtSUMO1(GG) and AtSCE1a (pACYCDuet-AtSCE1-AtSUMO1), the AtSUMO1 and AtSCE1a coding sequence fragments were separately cloned into the 2 multiple cloning sites of the expression vector pACYCDuet-1 (Novagen, Germany). The plasmid pETSAE1a2, which expresses AtSAE1a and AtSAE2, was kindly provided by Dr. Andreas Bachmair [77]. To construct the plasmid for DnSIZ1 expression, we generated the *pCDFDuet-1-Flag-DnSIZ1* and *pCDFDuet-1-Flag-DnSIZ1*^*C380A*^ constructs, the substitution of single amino acid was performed using the MutantBEST Kit (TaKaRa, Japan). The fragment was inserted into the *BamH*I/*Sac*I sites of multiple cloning site 1 of the expression vector pCDFDuet-1 (Novagen, Germany). Primer sequences for gene amplifications are listed in Additional file 2: Table S1.

### Analysis of expression profiles and stress treatments

The expression of *DnSIZ1* in *Dendrobium* vegetative and reproductive organs was investigated using semi-quantitative RT-PCR. RT-PCR experiments were also employed to study the transcript levels of *DnSIZ1* following plant exposure to different environment stress related treatments, including ABA, drought, cold, and high temperature. For ABA treatments, normally cultured *Dendrobium* seedlings were sprayed with 100 μmol/L ABA (Biosharp, China) and the treated plants were harvested at 3, 6, 12 and 24 h. Control samples were collected immediately prior to the treatments. For the drought treatments, seedlings were transferred to clean and dry filter paper and the detached leaves were placed in a growth chamber and harvested at 3, 6, 12 or 24 h after the transfer. To investigate temperature tolerance, normally cultured *Dendrobium* seedlings were placed at 4 °C for the cold treatment or 37 °C for the heat shock treatment for 3, 6, 12 or 24 h, while for the high temperature treatment seedlings were placed at 45 °C for 1, 2, 4 or 6 h. The wounding treatment involved slicing off the 1 cm leaf apex with a sterile razor blade: the area 1 cm from the excised site was defined as W1 and regions at sequential 1 cm intervals adjacent to W1 were defined as W2, W3, and W4, respectively. The wound-treated plants were placed in the growth chamber, and leaf samples were collected after 48 h [78]. After all treatments, plant materials were immediately frozen in liquid N_2_ and stored at -80 °C until needed for RNA extraction. Total RNA samples were extracted and processed for semi-quantitative RT-PCR as described before [79]. Primer sequences for gene amplifications are listed in Additional file 2: Table S1. Each treatment was repeated with three biological replicates.

### RNA isolation and semi-quantitative RT-PCR

Total RNA was extracted from the roots, stems, leaves, flowers and flower buds of *Dendrobium* seedlings as described previously [80]. *Arabidopsis* total RNA was isolated from seedlings using TRIZOL reagent (Invitrogen, USA) according to the manufacturer’s instructions and then treated with DNase I (deoxyribonuclease I, Sigma, USA) at room temperature for 15 min and at 70 °C for 10 min.

For RT-PCR analyses, 2 μg sample of total RNA was used as a template for the first strand cDNA synthesis with M-MLV reverse transcriptase and an Oligo (dT)_18_ primer (TaKaRa, Japan). Specific gene expression levels were analyzed by semi-quantitative RT-PCR [79].

Primer pairs were designed using Primer Premier 5.0 (Premier, Canada). Primer sequences for gene amplifications are listed in Additional file 2: Table S1. Each PCR assay was repeated with three biological replicates, each of which had three technical replicates, giving nine measurements for each treatment/time point.

### Subcellular localization assay

The constructs encoding DnSIZ1:GFP or GFP alone were introduced separately into *Arabidopsis* wide-type by transformation, and T2 plants were used for subcellular localization analysis. Seedlings of *35S*:*GFP* and *35S*::*DnSIZ1*:*GFP #4* were grown vertically on MS agar plates. Roots of 7 day-old transgenic seedlings were stained with 10 μg/mL propidium iodide (PI, Sigma, USA) solution for 1 min, washed with sterilized water, placed on microscope slides in a drop of water and then imaged using confocal microscopy. Fluorescence analysis was performed using a Carl Zeiss LSM710 confocal laser microscope (LSM710, Carl Zeiss, Germany).

### ABA germination and flowering time assays

To investigate ABA sensitivity, plants of the different genotypes were grown in the same conditions and seeds were collected at the same time. For each comparison, seeds from WT, *siz1-2* and two transgenic lines (HB4, HB17) were planted in triplicate on plates containing MS medium with or without different concentrations of ABA (0.1, 0.2 and 0.5 μM). The plates were incubated at 4 °C for 3 days before being transferred to 22 °C under long day conditions. The percentage of seed germination was scored at indicated times, with germination defined as an obvious emergence of the radicle through the seed coat. Cotyledon greening was quantified at the time of cotyledon opening and epicotyl exposure as the percentage of seedlings with green cotyledons. The plates were scanned using an Epson perfection V200 photo scanner (Epson, Japan). Flowering time analysis was performed by counting the numbers of rosette leaves at the time of flowering, at least 15-20 plants were used to determine the flowering time of each genotype [31], vernalization treatment was at 4 °C for three weeks.

### *In vivo* analysis of SUMO conjugates

For the analysis of SUMO conjugates, 10 day-old seedlings grown on medium were shocked at a 42 °C heat stress for 30 min and the untreated seedlings were as a control. Plant tissues (0.1 g) were ground in grinding buffer (0.5 ml) (50 mM sodium phosphate pH 7.0, 200 mM NaCl, 10 mM MgCl_2_ and 10 % glycerol, one protease inhibitor cocktail tablet/10 ml), vibrated and placed for 10 min on ice, then centrifuged at the maximum speed at 4 °C for 10 min and kept the supernatant. Protein concentration was measured using the Bio-Rad Protein Assay reagent (Bio-Rad, USA), and proteins were separated by sodium dodecyl sulphate–polyacrylamide gel electrophoresis (SDS–PAGE), transferred to polyvinylidene difluoride membrane (Millipore, USA), probed with anti-SUMO1 polyclonal antibody (Abcam, UK) and goat anti-rabbit IgG (Sigma, USA), and detected with the ECL plus Western Blotting Detection system (KeyGEN, China) following the manufacturer’s instructions.

### *In vitro* SUMOylation assays

To reconstitute the SUMOylation system *in vitro*, pETSAE1a2 was co-transformed with pACYCDuet-AtSCE1-AtSUMO1 or pACYCDuet-AtSUMO1 into *Escherichia coli* BL21 (DE3) cells to generate competent cells [59, 60]. For the SUMOylation reaction, the *pCDFDuet-1-Flag-DnSIZ1* and *pCDFDuet-1-Flag-DnSIZ1*^*C380A*^ constructs were transformed into the above competent cells, respectively. Transformants were cultured in 5 ml of Luria-Bertani (LB) medium and shaken at 37 °C until the culture OD_600_ was 0.4-0.6, followed by the addition of 0.5 mM isopropyl β-D-thiogalactopyranoside (IPTG). After an approximately 12 h induction at 25 °C, cells were harvested from 300 μl of cell culture and 100 μl of 1 × SDS–PAGE sample buffer was added, followed by denaturation at 100 °C for 3 mins. Equal amounts of protein extracts were loaded onto each lane of an SDS–polyacrylamide gel. Electrophoresed proteins were blotted onto polyvinylidene fluoride (PVDF) membranes (Millipore, USA), and incubated with anti-FLAG antibody (Rabbit) (Sigma, USA) and sequentially with goat anti-rabbit IgG (Sigma, USA). Immunoblots were visualized by chemiluminescence (Millipore, USA).

### Sequences and bioinformatics analysis

Except for DnSIZ1, all of the protein sequences of the SIZ1 homologs identified by BlastP were downloaded from National Center for Biotechnology Information (NCBI) (http://www.ncbi.nlm.nih.gov/). Sequences of plant-specific SIZ proteins were then aligned using Clustal *X*2 [81]. A phylogenetic tree was constructed with the Maximum likelihood method [82], as described in MEGA 6.06 [83].

### Statistical analyses

Statistical analyses were performed using Microsoft Excel software (Microsoft Corp, USA) and a one-way analysis of variance (ANOVA). When significance was detected, sample means were separated using an LSD test at the *P* <0.05 level of significance with SPSS statistical software (SPSS, USA). The leaf length was measured using DIGIMIZER 3.2.1.0 (http://www.digimizer.com/).

## Additional files

Additional file 1: Figure S1.
**Coomassie brilliant blue staining of total protein.** Lanes 1, 3, 5, 7, 9 and 11, protein extracts before IPTG induction; lanes 2, 4, 6, 8, 10 and 12, protein extracts after IPTG induction. (TIFF 300 kb) Additional file 2: Table S1. Primers used in this study. (PDF 88 kb)

## Abbreviations

CaMV: Cauliflower mosaic virus
FLC: FLOWERING LOCUS C
FLD: FLOWERING LOCUS D
GA: gibberellin
GFP: green fluorescent protein
IPTG: isopropyl β-D-thiogalactopyranoside
LB: Luria–Bertani
MMS21: methyl methanesulfonate sensitive 1
MS: Murashige and Skoog
PIAS: protein inhibitor of activated STAT
RNAi: RNA interference
RT–PCR: reverse transcription-PCR
SA: salicylic acid
SAP: scaffold attachment factor A/B/acinus/PIAS
SIZ1: SAP and MIZ domain protein
STAT: signal transducers and activators of transcription
SUMO: small ubiquitin-related modifier
Ub: ubiquitin
Ubls: ubiquitin-like proteins
WT: wild type
