# Supplementary figures and images for: Functional characterization of DnSIZ1, a SIZ/PIAS-type SUMO E3 ligase from Dendrobium
Source: BMC Plant Biol. 2015 Sep 17;15:225. doi: 10.1186/s12870-015-0613-3 (PMC4574183; doi:10.1186/s12870-015-0613-3)

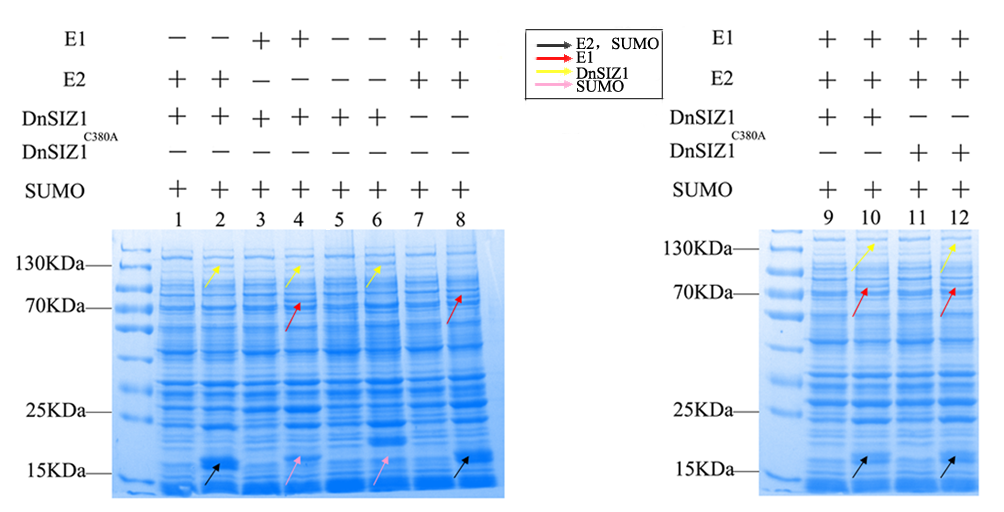

Supplement: Additional file 1: Figure S1. — Coomassie brilliant blue staining of total protein. Lanes 1, 3, 5, 7, 9 and 11, protein extracts before IPTG induction; lanes 2, 4, 6, 8, 10 and 12, protein extracts after IPTG induction. (TIFF 300 kb) [file 12870_2015_613_MOESM1_ESM.tiff]
