# Supplementary material for: Functional characterization of DnSIZ1, a SIZ/PIAS-type SUMO E3 ligase from Dendrobium
Source: BMC Plant Biol. 2015 Sep 17;15:225. doi: 10.1186/s12870-015-0613-3 (PMC4574183; doi:10.1186/s12870-015-0613-3)
Supplement: Additional file 2: Table S1. — Primers used in this study. (PDF 88 kb) [file 12870_2015_613_MOESM2_ESM.pdf]

| **Supplementary Table S1** Primers used in this study. | |
|---|---|
| Primer used for RT-PCR | |
| *DnSIZ1*-F | TGATTCCATTCTGTTACCTTCAGGTGCA |
| *DnSIZ1*-R | TGATGCGGTCTCATACCTTGGTTTCTGT |
| *siz1*-2-F | GAGGGATCGTCTCTACCAAATG |
| *siz1*-2-R | CTCAGTGGAGCATGTAATCGTC |
| *18sRNA*-F | GGTTCGCTGCTCGTGACTCT |
| *18sRNA*-R | CAGGCACCGCTTATTTTTACA |
| *Actin*-F | CTACGAGCAGGAACTCGAGA |
| *Actin*-R | GATGGACCTGACTCGTCATAC |
| *AtSIZ1*-F (full length) | ATGGATTTGGAAGCTAATTGTAAGG |
| *AtSIZ1*-R (full length) | TTACTCAGAATCCGAGTCAATGGAG |
| *DnSIZ1*-F (full length) | ATGGATTTGGCCATCAGTTGTAAGG |
| *DnSIZ1*-R (full length) | ATCAGAATCTGATTCCAAATTTATTGAGTG |
| Primer used for vector constructions | |
| pCAMBIA1300-221-*DnSIZ1*- | GGTACCCATGGATTTGGCCATCAGTTG (*Kpn* I) |
| pCAMBIA1300-221-*DnSIZ1*- | GCCCTCACTAGTAGATAAGACTTGACCATTGTC(*Spe* I) |
| pBEGFP-*DnSIZ1*-F | GGGGTACCATGGATTTGGCCATCAGT(*Kpn* I) |
| pBEGFP-*DnSIZ1*-R | CGGGATCCCGATCAGAATCTGATTCC (*Bam*H I) |
| CDF-Flag-R | ATAGGATCCAATGGATTTGGCCATCAGTTG(*Bam*H I) |
| CDF-Flag-F | ATAGAGCTCCCCTA CTTATCGTCGTCATCCTTGTAATC<br>ATCAGAATCTGATTCCAAATTTATT(*Sac* I) |
| CDF-Flag-Mu-R | AAGCCTGCCATTCACATGGGCTGT |
| CDF-Flag-Mu-F | AAACCTACCAGCTATCTTCATTCTAGACCCAC |
